# The Effects of Biofortified Cowpeas on Iron, Glucose, and Lipid Metabolism in Rats Fed a High‐Fat/High‐Sugar Diet

**DOI:** 10.1002/mnfr.70392

**Published:** 2026-01-21

**Authors:** Ana Paula Ribeiro Gaspar, Cíntia Tomaz Sant' Ana, Mariana Grancieri, Ana Paula Carvalho de Moura Cruz, Leone Soromenho Viana, Eduardo Lorencetti Fornazier, Marisa da Silva Corrêa, Henrique Jordem Venial, Jankerle Neves Boeloni, Neuza Maria Brunoro Costa

**Affiliations:** ^1^ Postgraduate Program in Food Science and Technology, Agricultural Sciences and Engineering Center Federal University of Espírito Santo Alegre Espírito Santo Brazil; ^2^ Department of Pharmacy and Nutrition Center of Exact, Natural and Health Sciences Federal University of Espírito Santo Alegre Espírito Santo Brazil; ^3^ Department of Veterinary Medicine, Agricultural Sciences and Engineering Center Federal University of Espírito Santo Alegre Espírito Santo Brazil

**Keywords:** biofortification, carbohydrate metabolism, iron bioavailability, lipid metabolism

## Abstract

Iron deficiency anemia may affect carbohydrates and lipid metabolism. This study aimed to evaluate the iron bioavailability from biofortified cowpeas in the context of a high‐fat/high‐sugar diet and its relationship with carbohydrate and lipid metabolism. Forty‐eight Wistar rats were induced to anemia for 21 days, and during the repletion phase (35 days), the animals received diets containing 12 ppm of iron from ferrous sulfate or biofortified (BRS Aracê and BRS Tumucumaque) and a conventional (BRS Pajeú) cowpeas. The biofortified cowpea Aracê showed a hemoglobin gain similar to ferrous sulfate, improved crypt size in the colon, lowered insulin levels and area under the curve in the glucose tolerance test compared to ferrous sulfate (*p* < 0.05). Hepcidin levels were similar between the groups. The biofortified cowpeas increased the production of short‐chain fatty acids compared to ferrous sulfate (*p* < 0.05). Both biofortified and conventional cowpeas increased HDL‐c concentrations, reduced the total cholesterol/HDL‐c ratio, and decreased fecal triglyceride excretion (*p* < 0.05). The biofortification process favors beneficial metabolic changes in the iron, glucose, and lipid metabolism.

## Graphical abstract

Biofortified cowpea Aracê improved crypt size in the colon, lowered insulin levels. Biofortified cowpeas increased short‐chain fatty acids. Cowpeas increased HDL‐c concentrations, reduced the total cholesterol/HDL‐c ratio, and decreased fecal triglyceride excretion. The biofortification process favors beneficial metabolic changes in the iron, glucose and lipid metabolism.

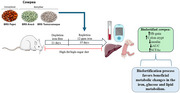

**Abbreviations**
GTT: glucose tolerance test
Hb: hemoglobin
HFHS: high‐fat/high‐sugar
HOMA‐IR: Homeostatic Model Assessment for Insulin Resistance
HRE: Hemoglobin Regeneration Efficiency
QUICKI: Quantitative Insulin Sensitivity Check Index
RBV: Relative Biological Value
SCFAs: short chain fatty acids

## 1 Introduction

Iron deficiency is the most prevalent nutritional deficiency worldwide and can be aggravated by an unhealthy high‐fat/high‐sugar diet with low proteins, vitamins, and minerals content [1]. This dietary pattern is directly linked to an increased risk of metabolic alterations, including changes in glucose, lipid, and iron metabolism [2, 3]. Much has been explored regarding the harmful effects of iron overload; however, iron deficiency also has a potential impact on glucose and lipid metabolism [4].

Metabolic alterations associated with the consumption of high‐fat and high‐sugar diets can lead to the dysregulated production of hepcidin, a key hormone that controls iron absorption. This imbalance may reduce iron stores by negatively affecting essential proteins involved in iron metabolism [5]. Limited iron availability impairs erythropoiesis and may consequently disturb glucose and lipid homeostasis [6]. Iron deficiency compromises glucose metabolism because iron is required for mitochondrial and oxidative enzymes. In its absence, glucose oxidation is reduced, leading to lower energy production. As a result, tissues such as muscle and liver exhibit decreased insulin‐dependent glucose uptake [7]. Additionally, iron deficiency upregulates HIF‐1α, which modulates glucose transporters (GLUT1 and GLUT3), thereby altering cellular glucose uptake [8]. Furthermore, iron plays a critical role in maintaining pancreatic β‐cell function and insulin secretion, suggesting that iron deficiency may contribute to the development of insulin resistance [7]. In addition, iron deficiency can result in alterations in the lipid metabolism, as iron is an essential cofactor of enzymes (acyl‐CoA oxidase, cytochrome c oxidase, aconitase), and its deficiency leads to reduced β‐oxidation of fatty acids, accumulation of triglycerides and lipids in liver and tissues, and decreased ATP production from fat [9]. Moreover, the iron deficiency‐induced increase in HIF‐1α downregulates the expression of PPAR‐α and CPT1A, key genes involved in fatty acid oxidation, thereby reducing lipid catabolism and promoting lipid accumulation [8].

Food biofortification is a strategy to reduce micronutrient deficiencies, particularly iron, zinc and vitamin A. Its main objective is to produce staple foods with higher micronutrient content to help reduce deficiencies in low‐income populations [10]. In the context of biofortified foods, cowpea (*Vigna unguiculata* L. Walp) is currently a potential target for iron biofortification and shows an increase of 30%–60% in iron content [11]. Cowpea is a highly nutritious food, containing relatively high concentrations of proteins, vitamins, dietary fibers, bioactive compounds, and minerals such as iron [12]. It is widely consumed in several underdeveloped countries, making it the primary source of dietary iron for these populations. Moreover, biofortified cowpeas may be an alternative source of iron for plant‐based diet consumers [13].

Previous studies conducted by our group demonstrated the iron bioavailability of biofortified cultivars in the context of a normocaloric diet, confirming the good iron bioavailability of biofortified cultivars under these conditions [12]. However, we know that the current dietary pattern is characterized by diets high in fats and simple carbohydrates, which promote the development of metabolic alterations and may interfere with the absorption and utilization of nutrients, including iron [14]. Therefore, considering the population's current dietary partner and the potential deleterious effects of iron deficiency and its secondary effects on metabolic alterations, studies that exploring viable and low‐cost alternatives to reduce the prevalence of these diseases are necessary. In this context, the objective of the present study was to evaluate the effects of biofortified cowpeas in the context of a high‐fat/high‐sugar diet and the potential benefits of these biofortified cowpeas on iron, glucose and lipid metabolism in rats.

## 2 Material and Methods

### 2.1 Material

The iron‐biofortified (BRS Aracê and BRS Tumucumaque), and the conventional cowpea cultivar (BRS Pajeú) were supplied by the Brazilian Agricultural Research Corporation (Embrapa Meio‐Norte, Piauí, Brazil) from harvest 2023. Cowpea grains were cooked, freeze‐dried, crushed, which were then added to the diets, according to previous study [12].

### 2.2 Composition Analysis of the Cowpeas

The protein, lipids, moisture, and ash were analyzed according to the Association of Official Analytical Chemists [15]. The insoluble and soluble fiber content were determined by the enzymatic gravimetric method using α‐amylase, amyloglucosidase, and protease for enzymatic hydrolysis (Total Dietary Fiber Kit, Megazyme). The total fiber content was quantified by the sum of soluble and insoluble fibers. Carbohydrate content was calculated by subtracting the concentration of fibers, proteins, lipids, ash, and moisture from 100.

Tannins were determined by the methodology by Price et al. [16], with absorbances measured at 500 nm (Multiskan GO, Thermo Scientific), and the results were expressed in milligrams of catechin equivalent per gram of dry sample (mg CE/g). The phytic acid content was analyzed with commercial kit (K‐PHYT Phytic Acid (Phytate)/ Total Phosphorus Kit, Megazyme). The phytate:iron molar ratio was determined considering the molecular weight of phytate (659.91 g/mol) and the atomic weight of iron (55.8 g/mol). The resistant starch content was determined using the commercial kit (Resistant Starch Assay Kit, Megazyme).

Iron was determined by flame atomic absorption spectrometry (GBC Scientific Equipment). The sample was digested with P.A. nitric acid in MARS 6 microwave equipment (CEM Corporation) [15].

For total phenolics quantification, an aqueous extract was used (80% v/v) [17]. The total phenolic compounds were determined using the Folin–Ciocalteu method, and the absorbance was measured at 760 nm (Multiskan Go, ThermoScientific, USA). The results were expressed as milligrams of gallic acid equivalents per gram of sample (mg GAE/g) [18].

### 2.3 Animal and Experimental Design

The study was approved by the Committee on Ethics in Animal Use of the Federal University of Espírito Santo, Brazil (Protocol n°. 013/2022). All experimental procedures were performed in accordance with the ethical principles for animal experimentation and Animal Research guidelines: the ARRIVE Guidelines.

A total of 48 male Wistar rats (*Rattus norvegicus*), 21‐day‐old were individually housed in stainless steel cages under a controlled temperature (23°C ± 1°C), with a 12‐hour light‐dark cycle, and ad libitum access to deionized water. Weight and food intake were monitored weekly. The sample size was determined considering a type I error probability (*α*) of 5% (*Z* = 1.96 for a 95% confidence interval) and the mean hemoglobin gain and variability reported by Sant'Ana et al. as reference values for the expected effect size [19, 20].

The iron bioavailability was conducted using a depletion/repletion methodology with modifications, in which iron depletion (iron‐free diet) occurred over 21 days, followed by repletion (diets with iron) for 35 days [21]. The animals received the AIN‐93G diet recommended for rodents [22]. The diets provided were high‐fat/high‐sugar (HFHS), containing 30% lipids (lard) and 30% sucrose. The diets were prepared to have the same iron content (12 ppm), derived from cowpeas (experimental diets) or ferrous sulfate—FeSO_4_ (control diet). Based on the iron concentration determined in cowpea flours, these flours were added to the diets in quantities sufficient to provide a dose of 12 ppm of iron. Thus, to prepare 1 kg of diet, 246.87 g, 178.75 g, and 175.93 g of Pajeú, Tumucumaque, and Aracê bean flours, respectively, were incorporated. The composition of experimental diets is presented in Table S1.

In the depletion phase, the animals received HFHS (*n* = 48) and were fed an iron‐free diet ad libitum to induce anemia. At the end of the depletion phase, hemoglobin concentration was determined using a commercial kit (Labtest) through blood samples collected after the tip of the animals' tails was sectioned. Then, at repletion phase, the animals were randomly assigned into four experimental groups with the same initial hemoglobin concentration and body weight among groups. Thus, in the repletion phase, the study comprised four groups (*n* = 12): HFHS (control group): HFHS + FeSO_4_; C group: HFHS + conventional BRS Pajeú; B‐T group: HFHS + biofortified BRS Tumucumaque; and B‐A group: HFHS + biofortified BRS Aracê.

During the repletion phase, diets were provided daily (18 grams per day). At the end of the repletion phase, hemoglobin levels were measured again. Weight gain and food consumption were calculated. Feed efficiency rate (FER) was determined by the ratio between weight gain and food consumption in the repletion phase.

On the last day of the experiment, after 12 h of fasting, the animals were anesthetized by intraperitoneal administration of 0.2 mL/100 g body weight of anesthetic solution with ketamine (37.5%), xylazine (25%) and saline solution (37.5%). Blood was collected by cardiac puncture, followed by centrifugation at 3000 rpm for 10 min at 4°C to obtain serum, and then stored at –80°C for further analysis. The liver and colon were collected and frozen in ‐80°C, and one portion immersed in 10% formaldehyde for histological analysis. The cecal content was also collected and stored at –80°C.

#### 2.3.1 Iron Bioavailability and Iron Metabolism Biomarkers

Hemoglobin (Hb) gain was calculated as the difference in hemoglobin concentration between the end of the depletion and repletion phases. The iron pool in hemoglobin was calculated using the following formulas, assuming the total blood volume to be 6.7% of body weight and the iron content in hemoglobin to be 0.335% [23]. Relative Biological Value (RBV), and Hemoglobin Regeneration Efficiency (HRE) were calculated according to the following formulas [24].

$$\begin{array}{cc} & \text{Initial}\;\text{Hb}\text{--}\text{Fe}\;(\text{mg})\\ & \;=[\text{initial}\;\text{weight}\;(g)\;x\;\text{initial}\;\text{Hb}\;(g/\text{dL})\;x\;6.7\times 0.335]/1000 \end{array}$$

$$\begin{array}{cc} & \text{Final}\;\text{Hb}\text{--}\text{Fe}\;(\text{mg})\\ & \;=[\text{final}\;\text{weight}\;(g)\;x\;\text{final}\;\text{Hb}\;(g/\text{dL})\;x\;6.7\times 0.335]/1000 \end{array}$$

$$\begin{array}{cc} & \text{HRE}\;(\%)\\ & \;=(\text{final}\;\text{Hb-Fe}\;(\text{mg})\text{--}\text{initial}\;\text{HB}\\ & \;-\text{Fe}\;(\text{mg})/\;\text{iron}\;\text{consumed}\;(\text{mg}))\times 100 \end{array}$$

$$\begin{array}{ccc} \text{RBV} & = & \left(\text{HRE}\;\text{from}\;\text{test}\;\text{group}\;(\%)/\text{HRE}\;\text{from}\;\text{control}\;\text{group}\;(\%)\right)\\ & & \times \;100 \end{array}$$

Ferritin and transferrin were determined in the serum using commercial kits (Bioclin, Brazil), according to the manufacturer's instruction. Iron was analyzed in the serum by atomic absorption spectrometry [15]. The hepcidin concentration was performed using liver homogenate with a commercial ELISA kit (Elabscience Rat Hepcidin—E‐EL‐R0500) following the manufacturer's instructions.

#### 2.3.2 Biochemical Analysis

Total cholesterol, high density lipoprotein (HDL‐c), triacylglycerol, and glucose were determined from the serum of the animals based on the colorimetric method using commercial kits (Bioclin, Brazil). The low‐density lipoprotein (LDL‐c) concentration was estimated by the Friedewald et al. [25].

#### 2.3.3 Insulin and Glucose Tolerance Test

The insulin analysis was performed using serum with a commercial ELISA kit (Elabscience Rat Insulin—E‐EL‐R2466) following the manufacturer's instructions. Fasting blood glucose was measured in the serum using a commercial kit (Analisa) according to the manufacturer's instructions. The HOMA‐IR (Homeostatic Model Assessment for Insulin Resistance) and QUICKI (Quantitative Insulin Sensitivity Check Index) indices were calculated using the following formulas [26, 27].

$$\text{HOMA-IR}=\left(\text{insulin}\left(\text{mU}/L\right)\;\text{x glicose}\left(\text{mg}/\text{dL}\right)\right)/405$$

QUICK=1/(loginsulin(μU/mL)+logglycemia(mg/dL))

For glucose tolerance test (GTT), at the end of the experiment, after 12 h of fasting, a 50% glucose solution was administered via the intraperitoneal cavity (2 g/kg of body weight). Blood glucose concentration was analyzed using blood obtained from an incision at the terminal part of the tail at 0, 30, 60, 90, and 120 min after glucose administration. Blood glucose levels were quantified using a glucometer (Accu‐Chek Active). The incremental area under the blood glucose curve (iAUC) was calculated from 0 to 120 min using the trapezoidal method by glucose values corrected by subtracting fasting glucose in each animal.

#### 2.3.4 Short Chain Fatty Acids (SCFAs) and Cecal pH Measurement

The SCFAs analysis was conducted on caecum content following the methodology proposed by Siegfried et al. [28]. Briefly, 100 mg of caecum feces was homogenized with calcium hydroxide and cupric sulfate to extract SCFAs. Quantification was performed via HPLC using a Dionex Ultimate 3000 Dual Detector HPLC system (Dionex Corporation) equipped with a Shodex RI‐101 refractive index detector maintained at 40°C. Separation of SCFAs was achieved on a Bio‐Rad HPX‐87H column (300 × 4.6 mm) (Phenomenex Inc.) maintained at 45°C. The analysis was conducted under the following conditions: 5 mmol/L sulfuric acid as the mobile phase, a flow rate of 0.7 mL/min, a column temperature of 45°C, and an injection volume of 20 µL. Stock solutions of standards were prepared using acetic, propionic, and butyric acids, with all SCFAs at a final concentration of 10 mmol/L.

Cecal content (100 mg) was diluted in distilled water (1:10), homogenized using a vortex, and the pH was measured with a digital pHmeter [29].

#### 2.3.5 Fecal and Liver Lipid Extraction

For fecal and liver lipid extract, feces and liver homogenate were used respectively. In the last week of the experiment, freshly excreted feces were collected, cleaned, dried, and ground. Subsequently, the fecal and liver lipid extraction was performed according to the methodology of Folch et al., using a chloroform and methanol solution [30]. The extracted lipids were resuspended with isopropyl alcohol, and the total cholesterol and triglyceride content was determined using a commercial kit (Labtest) following the manufacturer's recommendations.

#### 2.3.6 Histomorphometric Analysis of the Liver and Colon

Liver and colon samples were fixed in 10% formaldehyde and processed by the routine paraffin embedding method. Sections with a thickness of 4 µm were cut, mounted on slides, and stained with hematoxylin/eosin. Analyses were performed using an optical microscope (OPTICAM O500R), and histological images were captured with a 20X objective. For colon analysis, the crypt depth, crypt width, and thickness of the circular and longitudinal muscle layers were analyzed [31]. For liver analysis the presence of microvascular and macrovascular steatoses, and inflammation were evaluated [32]. For the analysis, 20 random fields from each animal were selected. The analyses were conducted using the ImageJ program (National Institute of Health, USA).

### 2.4 Statistical Analysis

Data normality was assessed using the Kolmogorov–Smirnov test. When data followed a normal distribution, they were analyzed by one‐way analysis of variance (ANOVA), and differences among group means were evaluated using Tukey's post hoc test. A significant level of 5% (*p* < 0.05) was adopted. All statistical analyses were performed using GraphPad Prism software, version 9.0 (GraphPad Software, San Diego, CA, USA).

## 3 Results

### 3.1 Composition Analysis of the Cowpeas

The iron content in biofortified cowpea beans was higher than in the conventional cultivar (*p* < 0.05, Table 1). All the cowpea beans showed similar concentrations of ash, lipids, total and insoluble dietary fiber, and phytate (*p* > 0.05, Table 1). The biofortified BRS Aracê had highest protein content, followed by BRS Tumucumaque and BRS Pajeú had the lowest values (*p* < 0.05, Table 1). The conventional Pajeú cowpea bean had higher levels of moisture, soluble dietary fiber, resistant starch, and total phenolic compounds (*p* < 0.05, Table 1). The tannins were detected in any beans and conventional cowpea BRS Pajeú had the highest phytate:iron ratio and biofortified BRS Aracê the lowest one.

**TABLE 1:** Nutritional composition of cowpeas (dry base).

	Conventional	Biofortified	
Compounds	BRS Pajeú	BRS Tumucumaque	BRS Arace
Ash (g/100 g)	3.86 ± 0.26^a^	3.86 ± 0.17^a^	4.17 ± 0.03^a^
Moisture (g/100 g)	2.46 ± 0.04^a^	1.89 ± 0.12^b^	1.64 ± 0.04^c^
Protein (g/100 g)	24.42 ± 0.29^c^	26.07 ± 0.40^b^	27.92 ± 0.66^a^
Lipid (g/100 g)	2.70 ± 0.02^a^	2.80 ± 0.14^a^	2.76 ± 0.10^a^
Digestible carbohydrates (g/100 g)	43.33	40.71	42.15
Total fiber (g/100 g)	23.23± 2.54^a^	24.68 ± 2.38^a^	21.37 ± 2.47^a^
Insoluble fiber (g/100 g)	17.41 ± 2.43^a^	22.65 ± 1.91^a^	19.91 ± 1.27^a^
Soluble fiber (g/100 g)	5.82 ± 0.11^a^	2.03 ± 0.47^b^	1.47 ± 1.20^b^
Tannins (mg CE/g)	nd	nd	nd
Phytic acid (g/100 g)	1.03 ± 0.05^a^	1.03 ± 0.03^a^	1.01 ± 0.13^a^
Iron (mg/kg)	48.61 ± 4.61^b^	67.17 ± 2.52^a^	68.21 ± 4.55^a^
Phytate:iron ratio	17.97	12.90	12.48
Resistant starch (g/100 g)	3.85 ± 0.37^a^	3.26 ± 0.25^b^	2.52 ± 0.26^c^
Total Phenolic (mg GAE/g)	77.44±2.07^a^	54.99±1.29^b^	51.01 ±0.48^b^

*Note*: Data expressed as the mean standard deviation (*n* = 3). Different letters in the same line mean statistical difference by Tukey test (*p* < 0.05).

Abbreviations: CE = catechin equivalent, GAE = galic acid equivalent, nd = not detected.

### 3.2 In Vivo Study

#### 3.2.1 Consumption and Weight Gain

There was no difference in food consumption and weight gain in the depletion and repletion phases (*p* > 0.05, Table 2). Thus, no difference was observed in FER and iron consumption between the groups (*p* > 0.05, Table 2).

**TABLE 2:** Weight gain, food consumption, food efficiency rate (FER), and iron consumption.

	Markers	HFHS	C	B‐T	B‐A
Depletion	Weight gain (g)	105.20 ± 20.01^a^	101.60 ± 15.63^a^	105.20 ± 17.73^a^	105.50 ± 13.91^a^
	Food consumption (g)	293.20 ± 50.54^a^	285.80 ± 31.82^a^	325.90 ± 27.27^a^	286.20 ± 42.92^a^
Repletion	Weight gain (g)	85.37 ± 21.36^a^	112.89 ± 40.21^a^	104.05 ± 36.95^a^	104.10 ± 31.91^a^
	Food consumption (g)	523.51 ± 35.51^a^	499.83 ± 35.58^a^	531.55 ± 60.62^a^	504.83 ± 34.49^a^
	FER	16.46 ± 4.03^a^	20.67 ± 5.53^a^	19.55 ± 6.26^a^	22.41 ± 7.52^a^
	Iron consumption (mg)	8.69 ± 0.68^a^	8.79 ± 0.63^a^	9.39 ± 1.01^a^	9.21 ± 1.06^a^

*Note*: Data expressed as the mean standard deviation (*n* = 12). Different letters in the same line mean statistical difference by Tukey test (*p* < 0.05).

Abbreviations: B‐A = HFHS + biofortified Aracê, B‐T = HFHS + biofortified Tumucumaque, C = HFHS + conventional Pajeú, FER = food efficiency rate, HFHS = HFHS + FeSO_4_.

#### 3.2.2 Iron Bioavailability

**FIGURE 1:**
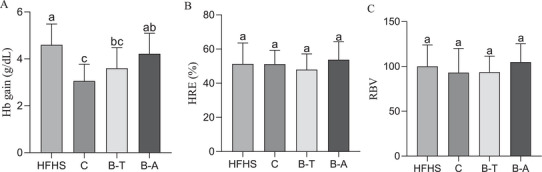
Iron bioavailability markers after consumption of experimental diets. (A) Hemoglobin gain; (B) %HRE analysis; (C) RBV analysis. Data expressed as the mean standard deviation (*n* = 12, biological replicates). Different letters mean statistical difference by Tukey test (*p* < 0.05). B‐A, HFHS + biofortified Aracê; B‐T, HFHS + biofortified Tumucumaque; C, HFHS + conventional Pajeú; Hb, hemoglobin; HFHS, HFHS + FeSO_4_; HRE, Hemoglobin Regeneration Efficiency; RBV, Relative Biological Value.

It was observed that the animals fed with conventional cowpea BRS Pajeú showed lower hemoglobin gain compared to ferrous sulfate group (*p* < 0.05, Figure 1A). The B‐A group had similar values to HFHS control group (*p* > 0.05, Figure 1A). Despite B‐T group showed similar values to B‐A, its values were similar to conventional cowpea too (*p* > 0.05, Figure 1A). There was no statistical difference in %HRE and RBV between the groups (*p >* 0.05, Figure 1B,C).

#### 3.2.3 Iron Metabolism Biomarkers

The concentrations of transferrin and serum iron were similar between the groups (*p* > 0.05, Table 3). However, the B‐T group had lower ferritin concentrations compared to the HFHS group (*p* < 0.05, Table 3). Hepcidin levels were similar between the groups (*p* > 0.05, Table 3).

**TABLE 3:** Iron metabolism markers, lipid profile, and fecal and liver lipid extraction after consumption of experimental diets.

Markers	HFHS	C	B‐T	B‐A
Ferritin (mg/dL)	113.80 ± 25.05^a^	65.15 ± 64.18^ab^	46.62 ± 13.00^b^	100.60 ± 23.53^ab^
Transferrin (mg/dL)	110.70 ± 7.22^a^	114.60 ± 7.84^a^	113.50 ± 5.64^a^	108.20 ± 4.61^a^
Serum iron (µg/dL)	60.21 ± 18.83^a^	57.34 ± 28.97^a^	43.20 ± 9.93^a^	64.79 ± 17.03^a^
Hepcidin (pg/mL)	22 236.36 ± 7319.54^a^	21 859.72 ± 4533.64^a^	18 686.36 ± 2244.69^a^	23 719.44 ± 5590.99^a^
TC	55.45 ± 13.24^a^	53.75 ± 8.96^a^	54.42 ± 6.62^a^	56.42 ± 7.28^a^
HDL‐c	18.82 ± 5.36^b^	22.58 ± 3.53^ab^	21.75 ± 2.80^ab^	23.92 ± 2.88^a^
LDL‐c	25.86 ± 8.47^a^	24.23 ± 5.79^a^	21.58 ± 5.15^a^	24.72 ± 5.28^a^
TG	54.64 ± 14.94^ab^	34.92 ± 15.47^b^	62.00 ± 13.84^a^	55.25 ± 17.90^a^
TC/HDL‐c	3.03 ± 0.21^a^	2.40 ± 0.27^b^	2.53 ± 0.28^b^	2.38 ± 0.19^b^
LDL‐c/HDL‐c	1.41 ± 0.27^ab^	1.60 ± 0.77^a^	1.03 ± 0.24^b^	1.05 ± 0.23^ab^
Feces lipid excretion (%)	11.95 ± 6.35^a^	8.01 ± 2.50^a^	7.61 ± 1.88^a^	12.43 ± 5.92^a^
TC fecal (mg)	1224.00 ± 543.30^a^	712.50 ± 257.20^b^	815.70 ± 222.60^ab^	880.70 ± 250.70^ab^
TG fecal (mg)	1018.00 ± 257.10^a^	723.90 ± 168.30^b^	692.00 ± 87.64^b^	731.70 ± 123.70^b^
Liver lipid (%)	5.52 ± 1.59^a^	5.84 ± 1.27^a^	5.87 ± 1.10^a^	5.90 ± 2.18^a^
TC liver (mg)	82.71 ± 12.27^a^	89.62 ± 13.80^a^	92.39 ± 11.39^a^	92.39 ± 11.26^a^
TG liver (mg)	309.70 ± 124.40^a^	281.20 ± 93.13^a^	314.80 ± 95.96^a^	314.80 ± 95.96^a^

*Note*: Data expressed as the mean standard deviation (*n* = 12). Different letters in the same line mean statistical difference by Tukey test (*p* < 0.05).

Abbreviations: B‐A = HFHS + biofortified Aracê, B‐T = HFHS + biofortified Tumucumaque, C = HFHS + conventional Pajeú, HDL‐c = high‐density lipoprotein, HFHS = HFHS + FeSO_4_, LDL‐c = low‐density lipoprotein, TC = total cholesterol, TG = triglycerides.

#### 3.2.4 Insulin and Glucose Tolerance Test (GTT)

**FIGURE 2:**
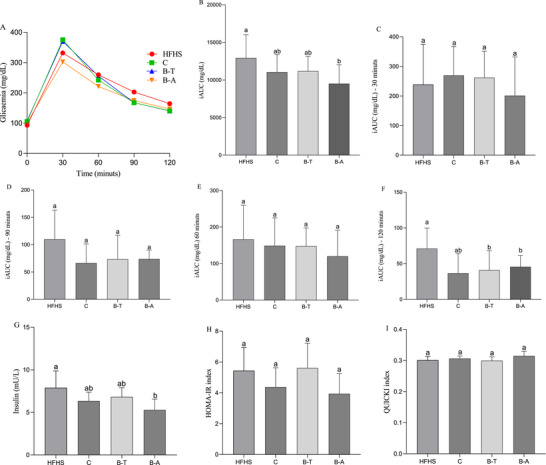
Markers of glucose metabolism in rats fed with conventional and biofortified cowpeas and high‐fat/high‐sugar diet. (A) Glycaemia of animals over 120 min; (B) Final incremental area in the glucose tolerance test; (C) Incremental area in the glucose tolerance test at 30 min; (D) Incremental area in the glucose tolerance test at 60 min; (E) Incremental area in the glucose tolerance test at 90 min; (F) Incremental area in the glucose tolerance test at 120 min; (G) Insulin level; (H) HOMA‐IR index; (I) QUICKI index. Data expressed as the mean standard deviation (*n* = 12, biological replicates). Different letters mean statistical difference by Tukey test (*p* < 0.05). B‐A, HFHS + biofortified Aracê; B‐T, HFHS + biofortified Tumucumaque; C, HFHS + conventional Pajeú; HFHS, HFHS + FeSO_4_; HOMA‐IR, homeostatic model assessment for insulin resistance; iAUC, area under curve; QUICKI, quantitative insulin sensitivity check index.

The B‐A group showed the smallest incremental area in the glucose tolerance test (*p* < 0.05, Figure 2A,B). At times 30, 60, and 90 min, no difference was observed between the groups (*p* > 0.05, Figure 2C–E). At time 120 min, the biofortified beans (B‐T and B‐A) showed a smaller incremental area compared to the HFHS group (*p* < 0.05, Figure 2F), however, conventional cowpea (C group) had similar values then control HFHS group and biofortified groups (*p* > 0.05, Figure 2).

Insulin levels were lower in the B‐A group compared to the HFHS group (*p* < 0.05, Figure 2G). There was no difference in the groups in relation to the HOMA‐IR and QUICKI indices (*p* > 0.05, Figure 2H,I).

#### 3.2.5 Short Chain Fatty Acids (SCFAs) and Cecal pH Measurement

**FIGURE 3:**
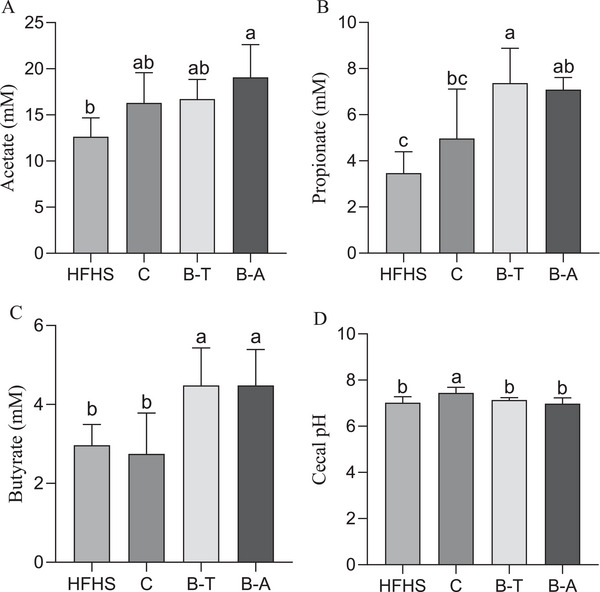
Short chain fatty acids and cecal pH after consumption of experimental diets by rats fed with cowpeas and high‐fat/high‐sugar diet. (A) Cecal acetate levels; (B) Cecal propionate levels; (C) Cecal butyrate levels; (D) Cecal pH. Data expressed as the mean standard deviation (*n* = 12, biological replicates). Different letters mean statistical difference by Tukey test (*p* < 0.05). B‐A, HFHS + biofortified Aracê; B‐T, HFHS + biofortified Tumucumaque; C, HFHS + conventional Pajeú; HFHS, HFHS + FeSO_4_.

The animals fed with Aracê cowpea had a higher acetate production compared to the HFHS control group (*p* < 0.05, Figure 3A). Propionate showed higher concentrations in the B‐T group (*p* < 0.05, Figure 3B), and the HFHS group had the lowest values. The butyrate concentration was higher in the groups with biofortified cowpea beans (B‐T and B‐A) (*p* < 0.05, Figure 3C). Cecal pH was higher in the conventional group (C) than other groups (*p* < 0.05, Figure 3D).

#### 3.2.6 Lipid Profile, and Fecal and Liver Lipid Extraction

Total cholesterol and LDL‐c did not show statistical difference between groups (*p* > 0.05, Table 3). The B‐A group presented higher HDL‐c concentrations when compared to the HFHS group (*p* < 0.05, Table 3). Cowpeas presented a lower TC/HDL‐c ratio (*p* < 0.05, Table 3). The C group presented lower triglyceride ​​(*p* < 0.05, Table 3).

There was no difference in lipid excretion in feces and liver lipid content (*p* > 0.05, Table 3). The conventional cowpea group (C) had lower total cholesterol concentration in feces (*p* < 0.05, Table 3), and all cowpeas (C, B‐T, B‐A) reduced fecal triglycerides compared to the HFHS group (*p* < 0.05, Table 3).

#### 3.2.7 Histomorphometric Analysis of the Liver and Colon

**FIGURE 4:**
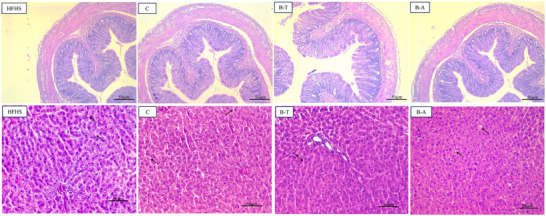
Representative pictures of colon and liver morphology of the experimental groups. B‐A, HFHS + biofortified Aracê; B‐T, HFHS + biofortified Tumucumaque; C, HFHS + conventional Pajeú; HFHS, HFHS + FeSO_4_.

There was no observed difference in circular and longitudinal muscle layer and crypts width between the groups (*p* > 0.05, Table 4). However, the biofortified bean Arace had an increase in crypts length compared to HFHS group (*p* < 0.05, Table 4 and Figure 4).

**TABLE 4:** Histomorphometric analysis of colon and liver after consumption of experimental diets.

Markers	HFHS	C	B‐T	B‐A
Circular muscle layer (µm)	24.51 ± 2.53^a^	27.34 ± 3.41^a^	29.50 ± 2.87^a^	28.68 ± 3.22^a^
Longitudinal muscle layer (µm)	61.38 ± 10.70^a^	65.21 ± 14.92^a^	69.38 ± 8.80^a^	62.83 ± 4.32^a^
Crypts length (µm)	80.04 ± 2.71^c^	100.54 ± 5.68^b^	104.47 ± 3.05^ab^	109.13 ± 4.09^a^
Crypts width (µm)	13.20 ± 1.31^a^	13.21 ± 1.29^a^	15.02 ± 1.88^a^	14.55 ± 1.65^a^
Macrovascular steatosis	0.33 ± 0.52^a^	0.17 ± 0.41^a^	0.00 ± 0.00^a^	0.00 ± 0.00^a^
Microvascular steatosis	1.33 ± 1.03^a^	1.67 ± 0.82^a^	0.50 ± 0.55^a^	1.17 ± 0.98^a^
Liver inflammation	nd	nd	nd	nd

*Note*: Data expressed as the mean standard deviation (*n* = 6). Different letters in the same line mean statistical difference by Tukey test (*p* < 0.05).

Abbreviations: B‐A = HFHS + biofortified Aracê, B‐T = HFHS + biofortified Tumucumaque, C = HFHS + conventional Pajeú, HFHS = HFHS + FeSO_4_, nd = not detected.

There was no difference in the level of hepatic steatosis between the groups, and there were no signs of inflammation in the liver (*p* > 0.05, Table 4 and Figure 4).

## 4 Discussion

The biofortified cowpea beans showed an increase of approximately 39% in the content of iron compared to the conventional cultivar, as expected in the biofortification program goal. However, more important than just the iron content is the need to assess the bioavailability of this mineral, since beans are a complex matrix containing various other components that can interfere with its bioavailability [12]. The levels of phytic acid and tannins, which are iron‐complexing compounds, were similar between the beans. However, phenolic compounds and fibers can also interfere with iron absorption, reducing its bioavailability [33]. The biofortified cowpea beans presented lower levels of soluble fiber, resistant starch, and total phenolic compounds compared to the conventional variety, but these differences were not sufficient to result in changes in iron bioavailability, since the iron bioavailability indices in the present study were similar between conventional and biofortified beans.

Based on the results of our vivo study, it was found that all the cowpeas lead to similar weight gain to the control group. The biofortified cowpea Aracê was more efficient in promoting hemoglobin gain, maintaining hemoglobin concentrations similar to those of ferrous sulfate, while the conventional cowpea bean showed lower hemoglobin gain. Thus, indicating that the biofortified cowpea Arace was more effective in increasing hemoglobin than the conventional cowpea bean in the context of high‐fat/high‐sugar diet. Adequate iron absorption is an important step in preventing anemia, as the amount of available iron is one of the key stages for it to be subsequently utilized in the body [5]. Furthermore, by evaluating the bioavailability indices HRE and RBV, which reflect hemoglobin formation based on the amount of iron consumed, we found that all cowpea beans were similar to the ferrous sulfate control group. The iron bioavailability of cowpea beans was similar to the ferrous sulfate treatment, with the results of iron metabolism biomarkers (transferrin, serum iron, hepcidin), which did not show alterations with bean consumption.

Serum iron concentrations did not show a statistically significant difference between the groups, as shown in the Table 3. The variable that did show a difference was hemoglobin gain, with conventional beans resulting in lower hemoglobin gain. Hemoglobin gain reflects an absolute physiological response (how much the hemoglobin level increased), whereas HRE is an index that expresses the proportion of absorbed iron that was effectively used to regenerate hemoglobin. Thus, even with more iron absorbed and a greater increase in hemoglobin, the utilization efficiency can remain the same [24].

In contrast, a recent study reported higher iron bioavailability in the biofortified Tumucumaque cowpea compared with a control normocaloric diet [12]. This discrepancy may stem from the different diet composition and health status of the animals, as the HFHS diet used in our study may negatively affect iron metabolism and interfere with the iron bioavailability of this biofortified cowpea. Sonweber et al. reported that mice fed a high fat diet developed iron deficiency due to a reduction in iron absorption [34]. Thus, comparisons among studies that investigate iron bioavailability in biofortified foods should take into consideration the diet composition and metabolic state of the animals.

The bioavailability of iron may be related to the increased production of the SCFAs butyrate and propionate in the B‐A group, which may have contributed to the greater Hb gain in this group. Iron deficiency has a strong regulatory effect on SCFA levels, particularly butyrate. Rats fed an iron‐deficient diet had significantly lower levels of butyrate and propionate accompanied by a decrease in major SCFA‐producing species compared with rats fed normal diet [35]. SCFAs, resulting from the fermentation of soluble fibers, promote nutrient absorption, including iron, by reducing intestinal pH, which favors the more soluble form of iron [36]. Furthermore, these SCFAs promote the integrity of the intestinal barrier, providing a larger surface area for iron absorption [37]. This information is congruent with our findings of greater crypt thickness in the B‐A group that may have contributed to a larger surface area for iron absorption. Although most iron absorption occurs in the duodenum, a considerable amount can also be absorbed in the colon [38]. Moreover, the SCFA propionate has been shown to increase levels of HIF‐2α (Hypoxia‐Inducible Factor‐2 Alpha), which regulates the expression of divalent metal transporter 1 (DMT‐1), duodenal cytochrome b (DcytB), and ferroportin genes, which improves iron metabolism [39, 40].

It is noteworthy that the conventional cowpea diet had a higher inclusion of cowpeas due to the lower iron content of this cultivar. Although all diets contained the same fiber content, the conventional Pajeú beans had more soluble fiber. Despite this, the biofortified beans resulted in greater SCFA production and improvements in crypt size in the colon, further highlighting the positive potential of biofortified cowpea beans. Additionally, the rats fed the biofortified cowpeas had lower intestinal cecal pH than those fed the conventional cowpea beans, despite the latter having a higher soluble fiber content, which is in contrast with previous findings that indicate that higher concentration of soluble fibers promote SCFA production and reduce intestinal pH. A lower pH favors the reduction of iron to its Fe^2^^+^ form, which can be absorbed by enterocytes. These findings suggest that biofortified cowpea beans may be beneficial for cecal production of SCFAs. However, further research is needed to determine the pathways and compounds responsible for this increase in SCFAs production and reduction of intestinal pH.

Iron bioavailability is an important factor that can impact lipid profiles, as iron is an essential micronutrient in lipoprotein metabolism, and the consumption of HFHS diets can alter lipid metabolism [9, 41]. In our study, the cowpea beans were able to promote some changes in the lipid profile, with the biofortified Aracê bean increasing HDL‐c levels compared to the HFHS group. Additionally, all the beans reduced the atherogenicity of diets, as evidenced by the reduction in the TC/HDL‐c ratio. Although fecal lipid excretion did not differ between groups, cowpea beans reduced triglyceride levels in the feces. However, this did not result in higher levels of lipids and triglycerides in the liver. Complementing these findings, no presence of fatty liver steatosis or liver inflammation was observed, demonstrating that the lower excreted triglyceride content was not accumulated in the liver. Compounds present in cowpea beans, such as soluble fibers and resistant starch, may contribute to reduced cholesterol absorption; however, this was not observed in the present study. Furthermore, SCFAs can suppress hepatic cholesterol synthesis. However, despite the higher production of these compounds in the groups that consumed biofortified cowpea beans, no effect on total cholesterol was observed.

The consumption of HFHS diets induces the development of glucose metabolism disorders, and iron deficiency may exacerbate it [4]. The consumption of biofortified cowpea Aracê resulted in lower insulin levels compared to the control group in the context of a HFHS diet. These results are similar with the glucose tolerance test, where the group consuming Aracê cowpea showed a lower incremental area under the curve. Although there were no differences between the groups at 30, 60, and 90 min, all the cowpeas reduced the incremental area under the curve at 120 min. Soluble fibers and resistant starch are recognized for their properties of promoting slower digestion, which can result in lower glucose concentrations over time, and other alternative pathways can be activated by compounds present in beans, acting to reduce blood glucose, such as the production of SCFAs, improving insulin sensitivity [42, 43]. In our study, conventional cowpea had higher levels of soluble fibers and resistant starch, but this did not result in reduced blood glucose. Therefore, we suggest that the lower insulin and area under the curve in the Aracê group were related to other compounds present in the cowpea, such as bioactive peptides, phytochemicals, or products from alternative pathways that acted in alternative ways and not just in promoting slower digestion.

A study conducted with black beans suggests that the SCFA butyrate may mediate the reduction of insulin levels [44]. In terms of glucose metabolism, butyrate and acetate stimulate glucagon‐like peptide 1 release, thereby indirectly influencing insulin secretion and glucose homeostasis. Increasing evidence also highlights the SCFA–G protein‐coupled receptor (GPCR) axis as a key player in pancreatic β‐cell function and the regulation of insulin secretion [45].

It is important to highlight the limitations of this study, as we know that there are differences in metabolism between rats and humans. Based on the results obtained, further research is suggested to evaluate different metabolic pathways and conduct more biomolecular analyses, as well as to study the effects of other compounds present in cowpea and their potential effects. However, the present study provides important information for future studies involving humans.

## 5 Conclusion

This study found that biofortified cowpeas promoted hemoglobin gain in anemic rats similarly to ferrous sulfate, even on a high fat/high sugar diet. Furthermore, consumption of Aracê variety increased HDL‐c and improved the body`s insulin response when exposed to high glucose levels. These results may be associated with increased production of SCFAs, especially butyrate and acetate, produced by animals that consumed Aracê cowpeas.

Although the bioavailability of iron from cowpea was comparable to that of ferrous sulfate, future human studies would be necessary to elucidate the possible benefits of biofortification on iron nutritional status and potential metabolic benefits of cowpea. Thus, the biofortification process causes changes in the nutritional composition of cowpeas, favoring beneficial metabolic changes in the organism of rats, such as iron, glucose, and lipid metabolism.

## Funding

This work was supported by the Fundação de Amparo à Pesquisa e Inovação do Espírito Santo [FAPES TO 477/2021, TO 567/2018, TO 689/2022, TO 1035/2022] and Coordenação de Aperfeiçoamento de Pessoal de Nível Superior [CAPES 001].

## Ethics Statement

The study was approved by the Committee on Ethics in Animal Use of the Federal University of Espírito Santo, Brazil (Protocol n°. 013/2022). All experimental procedures were performed in accordance with the ethical principles for animal experimentation and Animal Research guidelines: the ARRIVE Guidelines.

## Conflicts of Interest

The authors declare no conflicts of interest.

## Supporting information




**Supporting File**: mnfr70392‐sup‐0001‐SuppMat.docx.

## Data Availability

Data sharing not applicable to this article as no datasets were generated or analyzed during the current study.
