# Supplementary material for: The Effects of Biofortified Cowpeas on Iron, Glucose, and Lipid Metabolism in Rats Fed a High‐Fat/High‐Sugar Diet
Source: Mol Nutr Food Res. 2026 Jan 21;70(2):e70392. doi: 10.1002/mnfr.70392 (PMC12824469; doi:10.1002/mnfr.70392)
Supplement: Supplementary file 1 — Supporting File: mnfr70392‐sup‐0001‐SuppMat.docx. [file MNFR-70-e70392-s001.docx]

**Supplementary Material**

Title: The effects of biofortified cowpeas on iron, glucose and lipid metabolism in rats fed a high-fat/high-sugar diet.

**Table S1.** Composition of experimental diets (g/kg of diet).

|  | Repletion | | | |
| --- | --- | --- | --- | --- |
| Ingredients (g/kg) | HFHS | C | B-T | B-A |
| Albumin* | 200.00 | 120.95 | 138.95 | 135.59 |
| Dextrinized starch | 92.18 | - | 23.71 | 23.13 |
| Sucrose | 300.00 | 288.35 | 300.00 | 300.00 |
| Soybean oil | 70.00 | 63.33 | 65.00 | 65.14 |
| Lard | 230.00 | 230.00 | 230.00 | 230.00 |
| Cellulose | 57.32 | - | 13.19 | 19.71 |
| Mineral mix - iron free | 35.00 | 35.00 | 35.00 | 35.00 |
| Vitamin mix | 10.00 | 10.00 | 10.00 | 10.00 |
| L-cystine | 3.00 | 3.00 | 3.00 | 3.00 |
| Choline bitartrate | 2.50 | 2.50 | 2.50 | 2.50 |
| Cowpea** | - | 246.87 | 178.65 | 175.93 |
| Ferrous sulphate** | 0.0596 | - | - | - |
| Iron (mg/kg) | 16.81 | 17.59 | 17.67 | 18.26 |

*Purity of 77%. **quantity to provide 12 ppm of iron. HFHS: HFHS + FeSO₄; C: HFHS + conventional Pajeú; B-T: HFHS + biofortified Tumucumaque; B-A: HFHS + biofortified Aracê.
